# Resting-State Peripheral Catecholamine and Anxiety Levels in Korean Male Adolescents with Internet Game Addiction

**DOI:** 10.1089/cyber.2015.0411

**Published:** 2016-03-01

**Authors:** Nahyun Kim, Tonda L. Hughes, Chang G. Park, Laurie Quinn, In Deok Kong

**Affiliations:** ^1^ Keimyung University College of Nursing, Daegu, Korea.; ^2^ University of Illinois at Chicago, College of Nursing, Chicago, Illinois.; ^3^ Department of Physiology, Yonsei University Wonju College of Medicine, Wonju, Korea.

## Abstract

The purpose of this study was to compare the resting-state plasma catecholamine and anxiety levels of Korean male adolescents with Internet game addiction (IGA) and those without IGA. This cross-sectional comparative study was conducted with 230 male high school students in a South Korean city. Convenience and snowball sampling methods were employed, and data were collected using (1) participant blood samples analyzed for dopamine (DA), epinephrine (Epi), and norepinephrine (NE) and (2) two questionnaires to assess IGA and anxiety levels. Using SPSS 15.0, data were analyzed by descriptive analysis, χ^2^-tests, *t*-tests, and Pearson's correlation tests. The plasma Epi (*t* = 1.962, *p* < 0.050) and NE (*t* = 2.003, *p* = 0.046) levels were significantly lower in the IGA group than in the non-IGA group; DA levels did not significantly differ between the groups. The mean anxiety level of the IGA group was significantly higher compared with the non-IGA group (*t* =−6.193, *p* < 0.001). No significant correlations were found between catecholamine and anxiety levels. These results showed that excessive Internet gaming over time induced decreased peripheral Epi and NE levels, thus altering autonomic regulation, and increasing anxiety levels in male high school students. Based on these physiological and psychological effects, interventions intended to prevent and treat IGA should include stabilizing Epi, NE, and anxiety levels in adolescents.

## Introduction

Internet addiction (IA) is one of the most pervasive public health issues among youth worldwide. In Korea, almost 100 percent of adolescents access the Internet on a daily basis.^1^ This high rate of Internet use has been accompanied by an increase in IA.^2^ According to a recent survey performed by the Korean government, the IA rate is 11.7 percent among middle and high school students, the highest among all age groups in Korea.^1^ Internet game addiction (IGA) is a subtype of IA,^3^ and IGA has received more social and research attention than other subtypes such as using social networking services, viewing pornography, and online shopping.^4^ IGA has been subjected to so much scrutiny because it has more serious individual and social consequences than other pathological Internet activities. In Korea, gaming is the major purpose of Internet use among adolescent high-risk Internet users,^1^ and an increasing number of adolescents are considered to be at risk for IGA.^5^

People with IGA, defined as excessive or compulsive use of games that interferes with daily life, tend to isolate themselves from social contact and concentrate almost entirely on game activities.^3^ IGA and IA share features such as excessive and poorly controlled use of the Internet and impairments in daily living. In addition, the diagnostic criteria for IGA and IA are similar because, in general, they are adapted from the *Diagnostic and Statistical Manual of Mental Disorders* (DSM) criteria for pathological gambling.^6–8^ For these reasons, the terms IGA and IA have been used interchangeably in most previous studies. Although some argue that the conditions should be grouped together,^9^ demographic characteristics and clinical features of individuals with IGA and IA tend to differ. For example, IGA is more prevalent among males than females and poses a lower risk of depression than IA.^10–12^ Moreover, the fact that the Fifth Edition of the DSM (DSM-V) included Internet gaming disorder as a condition warranting further study^5^ highlights the significance of Internet gaming as distinct from the broader phenomenon of IA.

Although individuals with IGA have great difficulty controlling their excessive online gaming and IGA has been recognized as a potentially serious psychiatric problem,^4^ there is no standard definition or intervention for IGA at this time.^4,13,14^ To date, a number of studies have identified factors related to IGA. Most research has focused on personal and psychosocial risk factors,^15,16^ with stress being among the most significant psychosocial risk factors.^15,17^ Frequently, IGA is accompanied by other psychiatric problems such as depression, anxiety, or attention-deficit hyperactivity disorder,^16,18,19^ conditions also associated with stress.^17^ In many recent neurobiological studies, however, definite structural and functional changes have been identified in the limbic area and prefrontal cortex of the brain in Internet addicts.^3,20^ These studies indicate that repetitive and excessive Internet game use may alter the brain structure and functions underlying specific cognitive processes, resulting in cognitive control deficits that lead to IGA.^4,14^ Nevertheless, little is known about the physiological characteristics underlying IGA.

It is noteworthy that online gaming has been related to changes in salivary cortisol,^21^ physiological arousal,^22^ and shifts in heart rate variability^23,24^ during game playing. These adverse physiological changes have been observed even in the basal (nongaming) state among people with IGA. In a previous study, we identified greater basal-state plasma cortisol levels in excessive Internet game users compared to nonexcessive users.^25^ Findings from our and other physiological studies suggest that excessive Internet gaming use is associated with autonomic dysregulation,^23,26^ although results have been inconsistent.

Stress is recognized as a predisposing factor in most forms of addiction.^27,28^ Stress triggers numerous physiological changes^17^ and has been proposed as a likely mechanism underlying the IGA development.^21^ Despite the clear associations between stress and addiction, few studies have attempted to identify physiological stress responses to IGA. Although catecholamines are the first line of physiological response to stress, plasma catecholamine levels have not been measured in people with IGA.

Catecholamines, including dopamine (DA), norepinephrine (NE), and epinephrine (Epi), regulate stress-induced sympathetic activity. Normally stress responses help individuals adjust to external and internal stimuli by activating two major systems: the rapidly acting sympathetic adrenergic system (SAS) and the slower hypothalamic–pituitary–adrenal axis.^17,29^ The SAS releases catecholamines from sympathetic nerve endings and adrenal glands, and these chemicals work as a functional unit to cause “fight-or-flight” responses in emergencies.^30^ Although peripheral catecholamines are not permeable to the blood–brain barrier, circulating Epi and NE may also communicate with central dopaminergic and noradrenergic neurons through vagal afferent pathways.^29,31^ Therefore, inadequate responsiveness of the SAS can result in the development of various acute and chronic diseases, including addictions.^29,32^ For these reasons, catecholamines have been targeted to prevent and treat Internet gaming disorder.^13,33,34^

In the present study, we examined stress-induced physiological indicators in plasma catecholamines—that is, DA, Epi, and NE levels—in IGA and non-IGA subjects. Because anxiety is strongly associated with catecholamines in the central nervous system (CNS),^35,36^ we also examined anxiety levels as an indicator of emotional stress. In general, stress induces sympathetic responses by the release of catecholamines, and thus, prolonged stress responses could result in autonomic dysregulation. Therefore, we hypothesized that male adolescents with IGA would exhibit an alteration in plasma catecholamine levels and higher levels of anxiety than those not addicted to Internet gaming. We further hypothesized that catecholamine levels would be associated with self-reported anxiety levels.

## Methods

### Participants and procedures

Subjects were 15- to 18-year-old boys recruited from nine urban vocational high schools in Korea. Because male adolescents are more commonly addicted to Internet gaming^1,15^ and female sex hormones can affect regulation of addiction-related hormones such as DA, the study was limited to male students.^37^ Students with a diagnosed medical condition or those taking medications (e.g., β-blockers or sedatives) that might affect plasma catecholamine levels were also excluded. We used convenience and snowball sampling methods for recruitment. We visited each high school and obtained permission to explain the study to students. We then entered each classroom during a break time to explain the study's purpose and procedures and invite interested students to participate. To increase the sample size, we asked subjects recruited during this process to invite acquaintances who were Internet game users to accompany them to the data collection site, where they were screened for eligibility.

Data were collected in a public sports center. Each subject completed the two study questionnaires in a private room, and blood samples were drawn. Data were collected between 8:00 and 10:00 a.m. under similar conditions. All subjects fasted for 12 hours before blood sampling. They were asked to refrain from smoking, drinking caffeinated beverages, and Internet gaming for 24 hours before data collection and were encouraged to have enough sleep the night before data collection. This study was approved by the Institutional Review Board of Yonsei University Wonju College of Medicine. We obtained written informed consent from each subject and his legal guardian.

### Measures

#### Internet game addiction

To screen for IGA, we used the Online Game Addiction Scale for Adolescents, which was developed by the Korean Agency for Digital Opportunity and Promotion (KADO)^38^ based on previous IA scales, counseling data for Internet game addicts, and expert panel discussions. The scale has established reliability and validity and has been used to screen for IGA among Korean adolescents in national surveys. The scale consists of 20 items with response options ranging from 1 = “not at all” to 4 = “always” (scores = 20–80, with higher scores indicating greater IGA). The scale consists of three subscales: (1) game-oriented life (e.g., “I feel better while I am in the virtual gaming world than in real life”), (2) loss of tolerance and control (“I cannot control the number of hours that I play Internet games”), and (3) withdrawal and affective experience (“I feel anxious and nervous when I cannot play Internet games”). According to KADO, a score of 49 or above on the scale indicates high IGA risk, and a score of 38 or above indicates overuse and potential IGA risk that may cause some problems in daily life.^29^ The scale's Cronbach's alpha in the current study was 0.93. Based on IGA scores, subjects were assigned to the non-IGA or IGA group.

#### Plasma catecholamine levels

The three plasma catecholamines—DA, Epi, and NE—were assayed using blood samples. Each subject was instructed to lie quietly for 20 minutes before blood sampling. Venous blood (5 mL) was extracted using a heparin anticoagulation vacuum tube. Catecholamine levels were measured by high-performance liquid chromatography (Agilent 1200 series; Agilent Technology).

#### Anxiety levels

We measured anxiety using the Revised Children's Manifest Anxiety Scale (RCMAS),^39^ a 37-item self-report measure of anxiety for youth aged 6 to 19. The RCMAS includes 37 common anxiety symptoms (yes/no) grouped into three subscales that assess physiological anxiety, worry/oversensitivity, and social concern (e.g., “I am afraid of a lot of things,” “I am nervous,” and “I often worry about something bad happening to me”). The total scale score ranges from 0 to 37, with a score above 15 considered clinically significant. The scale's Cronbach's alpha in the current study was 0.89.

### Data analysis

Data were analyzed using SPSS 15.0. Means, standard deviations, frequencies, and percentages were used to summarize the subjects' demographic and Internet gaming-related characteristics. Data for DA, Epi, and NE were not normally distributed and were converted by logarithm to achieve normal distribution. Independent *t*-tests were used to compare plasma DA, Epi, and NE and anxiety levels in the two groups. Correlations between plasma catecholamine and anxiety levels were analyzed using Pearson's coefficient. A *p*-value of <0.05 was considered statistically significant.

## Results

Table 1 presents demographic and Internet gaming-related characteristics. The subjects' mean age was 16.63 ± 1.02 years and mean body mass index was 21.91 ± 3.69 kg/m^2^. Approximately 25 percent reported that they smoked cigarettes and/or drank alcoholic beverages. About two-thirds (68.3 percent) were from dual-earner families. Daily sleep time significantly differed in the non-IGA and IGA groups (χ^2^ = 5.616, *p* = 0.018). Weekly Internet gaming frequency (χ^2^ = 45.994, *p* < 0.001) and daily Internet gaming time (*t* =−7.332, *p* < 0.001) were significantly higher in the IGA group. Average duration of Internet gaming was 6.82 ± 2.38 years in the non-IGA group and 7.64 ± 2.42 years in the IGA group (*t* = −2.409, *p* = 0.017). Average IGA scores were nearly twice as high in the IGA group (46.05 ± 8.96) as in the non-IGA group (26.43 ± 4.94; *t* = −20.708, *p* < 0.001).

**Table 1.** Comparison of Demographic and Internet Gaming-Related Characteristics of Non-IGA and IGA Group (*N* = 230)

		*Non-IGA group* (n = 112)	*IGA group* (n = 118)		
*Variables*	*Categories*	M ± SD *or* n *(percent)*		χ^2^ or t	p
Age (years)		16.63 ± 0.89	16.64 ± 1.13	−0.079	0.937
BMI (kg/m^2^)		22.06 ± 3.74	21.76 ± 3.65	0.602	0.548
Perceived academic performance	High rank	35 (31.3)	22 (18.7)	4.936	0.177
	Middle rank	58 (51.7)	72 (61.0)		
	Low rank	19 (17.0)	24 (20.3)		
Smoke cigarettes	No	84 (75.0)	87 (73.7)	0.049	0.473
	Yes	28 (25.0)	31 (26.3)		
Drink alcohol	No	86 (76.8)	87 (73.7)	0.288	0.351
	Yes	26 (23.2)	31 (26.3)		
Parents' employment status	Dual-income family	70 (62.5)	87 (73.7)	6.922	0.140
	Single-income family	36 (32.1)	24 (20.3)		
	Unemployed	6 (5.4)	7 (6.0)		
Sleep time (hours/day)	≤6	65 (58.0)	86 (72.9)	5.616	0.018
	>6	47 (42.0)	32 (27.1)		
Internet gaming frequency (days/week)	≤2	45 (40.1)	8 (6.8)	45.994	<0.001
	3–4	34 (30.4)	33 (28.0)		
	5–6	19 (17.0)	33 (28.0)		
	7	14 (12.5)	44 (37.2)		
Internet gaming time (minutes/day)		113.66 ± 82.53	227.29 ± 145.49	−7.332	<0.001
Duration of Internet gaming (years)		6.82 ± 2.38	7.64 ± 2.42	−2.409	0.017
IGA score		26.43 ± 4.94	46.05 ± 8.96	−20.708	<0.001

BMI, body mass index; IGA, Internet game addiction; kg/m^2^, kilograms per square meter.

In the non-IGA and IGA groups, the measured mean levels of DA were 56.95 ± 75.04 and 68.66 ± 82.75 pg/mL; Epi were 64.06 ± 94.50 and 48.35 ± 44.96 pg/mL, and NE were 412.95 ± 274.68 and 330.86 ± 178.67 pg/mL, respectively. Table 2 summarizes logarithmically converted plasma catecholamine and anxiety levels in the two groups. The IGA group's plasma Epi and NE levels were significantly lower compared with the non-IGA group (*t* = 1.962, *p* < 0.050 and *t* = 2.003, *p* = 0.046, respectively). The levels of plasma DA were higher for the IGA group, but not significantly so. Mean anxiety levels were significantly higher in the IGA group (*t* = −6.193, *p* < 0.001). No significant correlations were found between catecholamine and anxiety levels. However, IGA scores were significantly correlated with anxiety levels (*r* =0.452, *p* < 0.001), and daily Internet gaming time was slightly negatively correlated with plasma NE levels (*r* = −0.142, *p* = 0.032). Table 3 shows the correlation analysis results.

**Table 2.** Comparison of Plasma Catecholamine and Anxiety Levels of Non-IGA and IGA Groups (*N* = 230)

*Variables*	*Non-IGA group (*n = *112)* M ± SD	*IGA group (*n = *118)* M ± SD	t	p
Log DA	−3.28 ± 0.83	−3.13 ± 0.87	−1.370	0.172
Log Epi	−3.10 ± 0.74	−3.28 ± 0.68	1.962	<0.050
Log NE	−1.09 ± 0.66	−1.25 ± 0.55	2.003	0.046
Anxiety	21.67 ± 7.19	27.35 ± 6.13	−6.193	<0.001

DA, dopamine; Epi, epinephrine; NE, norepinephrine; Log, logarithm.

**Table 3.** Correlations Among Variables (*N* = 230)

*Variables*	*(1)*	*(2)*	*(3)*	*(4)*	*(5)*	*(6)*	*(7)*
(1) IGA score	1						
(2) Internet gaming time (minutes/day)	0.466^***^	1					
(3) Duration of Internet gaming (years)	0.177^**^	0.125	1				
(4) Log DA (pg/mL)	0.108	−0.112	0.057	1			
(5) Log Epi (pg/mL)	−0.107	−0.049	−0.001	−0.073	1		
(6) Log NE (pg/mL)	−0.105	−0.142^*^	−0.017	0.016	0.198^*^	1	
(7) Anxiety	0.452^***^	0.214^**^	0.074	−0.009	−0.021	−0.044	1

^*^ *p* < 0.05; ^**^p < 0.01; ^***^p < 0.001.

## Discussion

We investigated whether male adolescents with and without IGA differed in plasma catecholamine levels and self-reported anxiety levels. We found significant differences between mean plasma Epi and NE levels between the two groups. In the psychological domain, the mean anxiety score was markedly higher in the IGA group than in the non-IGA group. The IGA group reported an average of 7.64 years and 3.79 hours/day of Internet gaming (compared to 6.82 years and 1.89 hours/day in the non-IGA group). This excessive prolonged Internet gaming was likely related to the alterations in Epi and NE levels and the higher anxiety levels in the IGA group. These levels may have been associated with game-related stress because (1) Internet gaming has induced sympathetic activation in previous studies^21,23,24^ and (2) gaming activities have often been used as a stressor in studies measuring cardiovascular reactivity.^21,23^ Our results indicate that Internet game activity could itself cause physiological stress that, if continued over time, could result in IGA. Our plasma catecholamine results support the existence of Internet game-induced physiological stress.

Interestingly, plasma Epi and NE levels were lower in IGA than non-IGA subjects. These findings contrast with the elevated catecholamine levels associated with other psychiatric disorders such as post-traumatic stress syndrome.^40^ Moreover, our resting-state data showed patterns differing from those observed in most previous studies in which increased sympathetic tone occurred during and/or immediately after a gaming experiment.^21,22,24^ Our findings are partially consistent with those of a small case–control study where adolescents with IA showed lower serum NE levels than those without IA.^33^ In fact, ours is the first study suggesting the relevance of peripheral catecholamine to IGA. Although Epi—a major component of peripheral catecholamine—governs the fight or flight response, few studies have measured Epi responses.^41^ Exceptions are recent studies in which more attention has been given to Epi-determinant roles in short- and long-term stress-induced illnesses such as cardiovascular diseases, immune diseases, cancer, and psychiatric disorders.^31^

Based on our findings, we are unable to explain the underlying mechanisms for the reduced plasma catecholamine levels in the IGA group. However, the mechanisms are presumably associated with the “sensitization” or “downregulation” observed in the CNS of videogame addicts.^3,13^ Researchers have found that prolonged sympathetic stimuli suppress the brain's DA reward system. This suppression has been detected in the form of lower DA receptor (D2) availability and occupancy^3,42^ and lower DA transporter density in excessive videogame players.^43^ Downregulation is defined as a decrease in cellular components, including receptors and transporters, in response to external stimuli; this decrease reduces a cell's sensitivity to the stimuli. Some evidence exists for dopaminergic downregulation in the cellular receptor and transporter levels in Internet addicts,^42,43^ a phenomenon that has been well established in alcohol and other substance abusers.^44,45^

Downregulation may explain the reduced peripheral plasma catecholamine levels in our IGA group. Long-term stress induced by persistent Internet gaming may ultimately result in reduction of plasma Epi and NE levels due to receptor downregulation reflecting adaptive responses.^29^ At the CNS level, prolonged downregulation of specific receptors may contribute to cognitive impairment,^45^ which is thought to be a causal factor in IGA development.^4,14^ That is, stress-induced decreases in cognitive function could accelerate the transition from voluntary behavior to involuntary habitual behavior.^17^ However, we did not measure receptor downregulation related to catecholamines in this study. Future research should investigate the potential relationship between peripheral catecholamine levels and the density or occupancy of catecholamine receptors. With regard to DA, this catecholamine plays a key role in most psychological problems at the CNS level.^41^ However, the roles of DA in plasma, whose major sources include dietary intake and sympathetic nerves, are not well understood.^46^ Based on our data, peripheral DA, unlike DA in the brain, is not likely to be involved in IGA.

In addition to physiological mechanisms, stress responses involve psychological mechanisms. Anxiety is a major aspect of emotional distress and is associated with increased risk of addiction.^47^ Consistent with previous IA research, we found high anxiety levels in the IGA group.^19,33^ Zhang et al.^33^ argued that increased anxiety levels may be associated with altered NE functional activity in Internet addicts; however, we found no relationship between anxiety and catecholamine levels in our study. A possible explanation for this inconsistency is the use of different measures to assess anxiety (i.e., whereas Zhang et al. used the Self-Rating Anxiety Scale, we used the RCMAS). A second possible explanation is that at the CNS level, sustained activation of the NE system induced by prolonged stress exposure has been linked to increases in anxiety in animal models.^35,36^ However, at the peripheral level, physiological and psychological mechanisms may have been independently involved in IGA in our human subjects, notwithstanding the fact that Epi, NE, and anxiety levels differed between the IGA and non-IGA groups. On the other hand, we could not rule out the possibility that other factors mediated the relationship between plasma catecholamines and anxiety. Further research is needed to clarify how physiological and psychological mechanisms are independently involved in IGA and which factors mediate the relationship between plasma catecholamine and anxiety levels. Notably, we were unable to determine whether increased anxiety levels were a predisposing factor or a resulting symptom of excessive Internet gaming over time. In either case, anxiety should be a major focus in preventive and interventional strategies for adolescents engaging in excessive Internet gaming.

Given that previous literature has identified reduction of perceived stress as a major reason for excessive Internet use,^15,17^ our findings provide important new information. Based on our physiological and psychological results, we offer a tentative hypothesis about the relationship between stress and IGA, suggesting that antecedent psychological stress may combine with physiological stress induced by prolonged gaming activities to contribute to IGA development. Although more research is needed to identify additional physiological indicators and better understand the mechanisms underlying IGA, our results show the relevance of both physiological and psychological factors to IGA. These findings may contribute to identifying the pathophysiological mechanisms of IGA.

Our results have useful implications for IGA diagnosis and treatment, including the need for physiological and psychological assessment of IGA in youth. Currently, such assessments primarily focus on monitoring behavioral changes and self-report indicators. Furthermore, the findings have implications for the development of treatment strategies for adolescents with IGA. For example, interventions aimed at preventing and treating IGA in adolescents may need to focus on stabilizing Epi, NE, and anxiety levels.

Despite notable strengths of the study, two limitations should be considered. First, because our data are cross-sectional, causal associations among IGA, plasma catecholamine, and anxiety could not be determined. Longitudinal studies are needed to verify the study results. Second, IGA was measured using a self-report instrument. Subjects concerned about being stigmatized as addicts may have underreported their time spent in Internet gaming, resulting in underestimation of IGA.
